# Adapting beyond borders: Insights from the 19th Student Council Symposium (SCS2023), the first hybrid ISCB Student Council global event

**DOI:** 10.1093/bioadv/vbae028

**Published:** 2024-04-03

**Authors:** Syed Muktadir Al Sium, Estefania Torrejón, Sanjana Fatema Chowdhury, Rubaiat Ahmed, Aakriti Jain, Mirko Treccani, Laura Veschetti, Arsalan Riaz, Pradeep Eranti, Gabriel J Olguín-Orellana

**Affiliations:** Industrial Microbiology Research Division, BCSIR Chattogram Laboratories, Bangladesh Council of Scientific and Industrial Research, Chattogram 4220, Bangladesh; Metabolic Diseases Research Group, iNOVA4Health, NOVA Medical School, Faculdade de Ciências Médicas, Universidade NOVA de Lisboa, 1169-056 Lisbon, Portugal; Biological Research Division, BCSIR Dhaka Laboratories, Bangladesh Council of Scientific and Industrial Research (BCSIR), Dhaka 1205, Bangladesh; Laboratory of Population Genetics, Department of Biochemistry and Molecular Biology, University of Dhaka, Dhaka 1000, Bangladesh; Department of Biophysics, University of Delhi, New Delhi 110021, India; GM Lab, Department of Neurosciences, Biomedicine and Movement Sciences, University of Verona, 37134 Verona, Italy; GM Lab, Department of Neurosciences, Biomedicine and Movement Sciences, University of Verona, 37134 Verona, Italy; Infections and Cystic Fibrosis Unit, Division of Immunology, Transplantation and Infectious Diseases, IRCCS San Raffaele Scientific Institute, 20132 Milan, Italy; Precision Medicine Lab, National Center of Big Data and Cloud Computing, Peshawar 25000, Pakistan; Université Paris Cité, Inserm, T3S, F-75006 Paris, France; Centro de Investigación de Estudios Avanzados del Maule (CIEAM), Vicerrectoría de Investigación y Postgrado, Universidad Católica del Maule, Talca 3460000, Chile; Laboratorio de Bioinformática y Química Computacional, Departamento de Medicina Traslacional, Facultad de Medicina, Universidad Católica del Maule, Talca 3480094, Chile

## Abstract

**Summary:**

The 19th ISCB Student Council Symposium (SCS2023) organized by ISCB-SC adopted a hybrid format for the first time, allowing participants to engage in-person in Lyon, France, and virtually via an interactive online platform. The symposium prioritized inclusivity, featuring on-site sessions, poster presentations, and social activities for in-person attendees, while virtual participants accessed live sessions, interactive Q&A, and a virtual exhibit hall. Attendee statistics revealed a global reach, with Europe as the major contributor. SCS2023’s success in bridging in-person and virtual experiences sets a precedent for future events in Computational Biology and Bioinformatics.

**Availability and Implementation:**

The details of the symposium, speaker information, schedules, and accepted abstracts, are available in the program booklet (https://doi.org/10.5281/zenodo.8173977). For organizers interested in adopting a similar hybrid model, it would be beneficial to have access to details regarding the online platform used, the types of sessions offered, and the challenges faced. Future iterations of SCS can address these aspects to further enhance accessibility and inclusivity.

## 1 Introduction

The International Society for Computational Biology (ISCB) Student Council (SC) is an organization dedicated to fostering connections among students and early career researchers (ECRs) in the field of Computational Biology and Bioinformatics (Shome *et al.* 2016, Anupama *et al.* 2018, Parisi *et al.* 2019, Shome *et al.* 2019, Eranti *et al.* 2023). One of the flagship initiatives of ISCB-SC is the Student Council Symposium (SCS), which serves as an international platform for students worldwide to showcase their original work (Cuypers *et al.* 2016, Wilkins *et al.* 2016, Hassan *et al.* 2018, Parisi *et al.* 2019, Osorio-Mogollon *et al.* 2023). Together with its continental editions, the European SCS (Olguín-Orellana *et al.* 2021), Latin American SCS (Parra *et al.* 2014, Monzon *et al.* 2017, Parisi *et al.* 2019, Castillo-Vilcahuaman *et al.* 2020), African SCS (Souilmi *et al.* 2015, Rafael *et al.* 2017, Akurugu *et al.* 2020), and Asian SCS (Grover *et al.* 2023), their overarching mission is to nurture and empower the next generation of computational biologists by providing networking and soft skill development opportunities to their leading members and attendees, in general.

In the wake of the pandemic, we proudly hosted the first-ever hybrid SCS. This allowed participants from every corner of the globe to actively engage with the symposium while tailoring their experience to their preferences and schedules (Puccinelli *et al.* 2022). Virtual attendees immersed themselves in the symposium via a user-friendly platform, while those who preferred an in-person experience gathered in the charming city of Lyon, France.

Notably, SCS2023 marked a return to an in-person format after a series of disruptions caused by the COVID-19 pandemic. The symposium had been forced to adopt a fully virtual format from 2020 to 2022 (Kalia *et al.* 2020, Cuypers *et al.* 2021, Olguín-Orellana *et al.* 2021, Grover *et al.* 2023, Osorio-Mogollon *et al.* 2023). This return to in-person interactions was eagerly anticipated by the community, and it symbolized a face-to-face reunion of researchers and students, heightening scientific exchange after a hiatus of several years.

The decision to reintroduce an in-person component alongside the virtual offerings was made with meticulous consideration for safety and accessibility. It aimed to strike a balance between the advantages of face-to-face interactions and the flexibility that virtual platforms had brought to the symposium landscape (Ehteshami *et al.* 2022).

In this article, we will provide a comprehensive overview of the first hybrid and 19th ISCB Student Council Symposium (SCS2023). We will explore the symposium’s format, delve into the event’s highlights, challenges, and discuss its impact on the Computational Biology and Bioinformatics fields.

## 2 Format

One of the defining features of SCS2023 was its hybrid nature, which allowed attendees to tailor their participation to their preferences and circumstances. This approach aimed to maximize inclusivity and accessibility for a global audience.

### 2.1 In-person experience in Lyon, France

For those who preferred an in-person experience, SCS2023 hosted a face-to-face gathering in Lyon, France. The in-person component featured:

- On-site sessions: attendees in Lyon could participate in live sessions and discussions at the symposium venue. This provided an opportunity for on-site interactions with speakers and fellow attendees.
- Poster sessions: presenters had the chance to display their research in poster format, facilitating direct conversations and feedback from peers and experts.
- Social activities: following the main symposium, participants had the opportunity to expand their professional networks and make new connections in a relaxed environment.

### 2.2 Virtual attendance

Virtual attendees had the opportunity to engage in the symposium’s activities through a user-friendly online platform. This digital hub offered the following features:

- Live sessions: participants had convenient access to live talks, and panel discussions, ensuring they could fully partake in all aspects of the symposium’s content.
- Interactive Q&A: the virtual platform facilitated real-time interaction with presenters, allowing virtual attendees to ask questions and engage in discussions.
- Networking opportunities: attendees had the chance to connect with peers, presenters, and sponsors through dedicated virtual networking sessions, promoting collaboration and the exchange of ideas.
- Virtual exhibit hall: a virtual exhibit hall showcased cutting-edge tools, technologies, and resources within the field of Computational Biology and Bioinformatics, enabling attendees to explore and interact with exhibitors.

To obtain comprehensive information about the activities carried out, including details about the speakers, the duration of each talk, and the schedule distribution throughout the day, refer to the program booklet (Chowdhury *et al.* 2023).

### 2.3 Attendee statistics

**Figure 1.**
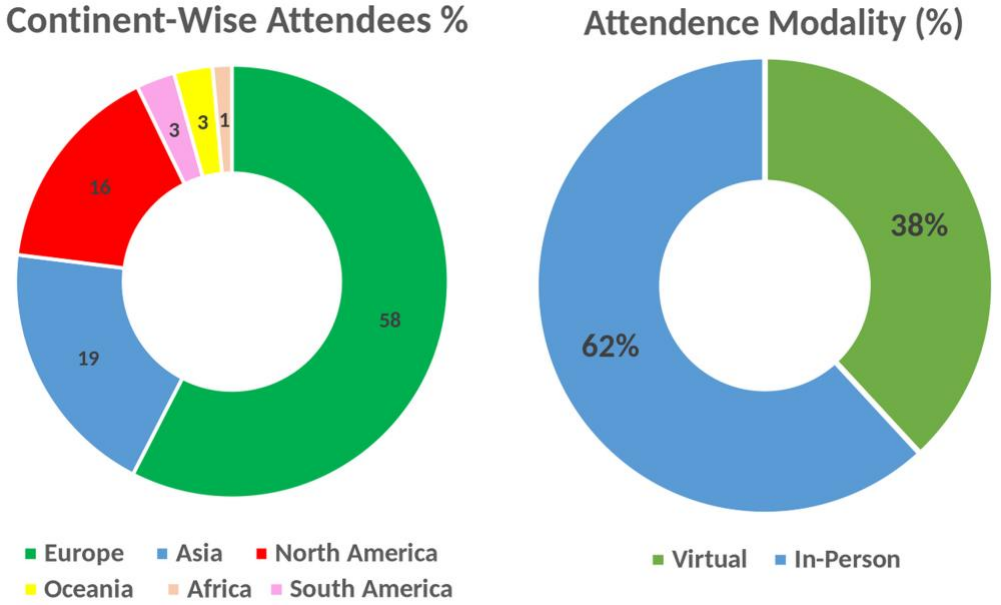
Attendee statistics of the Student Council Symposium 2023.

The attendee statistics for SCS2023 showcase a global reach, with participants hailing from diverse continents. The total number of participants was 139. Europe emerged as the predominant contributor, representing 58% of the attendees, followed by Asia at 19% and North America at 16%. South America and Oceania each constituted 3%, while Africa, though with a smaller representation at 1%. The participation of SCS2023 exhibits a balanced combination of virtual and in-person attendance. Approximately 38% of participants opted for a virtual experience, leveraging the symposium’s hybrid format to engage remotely. In contrast, a majority of 62% chose the in-person option, immersing themselves in the vibrant scientific atmosphere in Lyon, France. The attendance statistics of SCS2023 are shown in Fig. 1.

**Figure 2.**
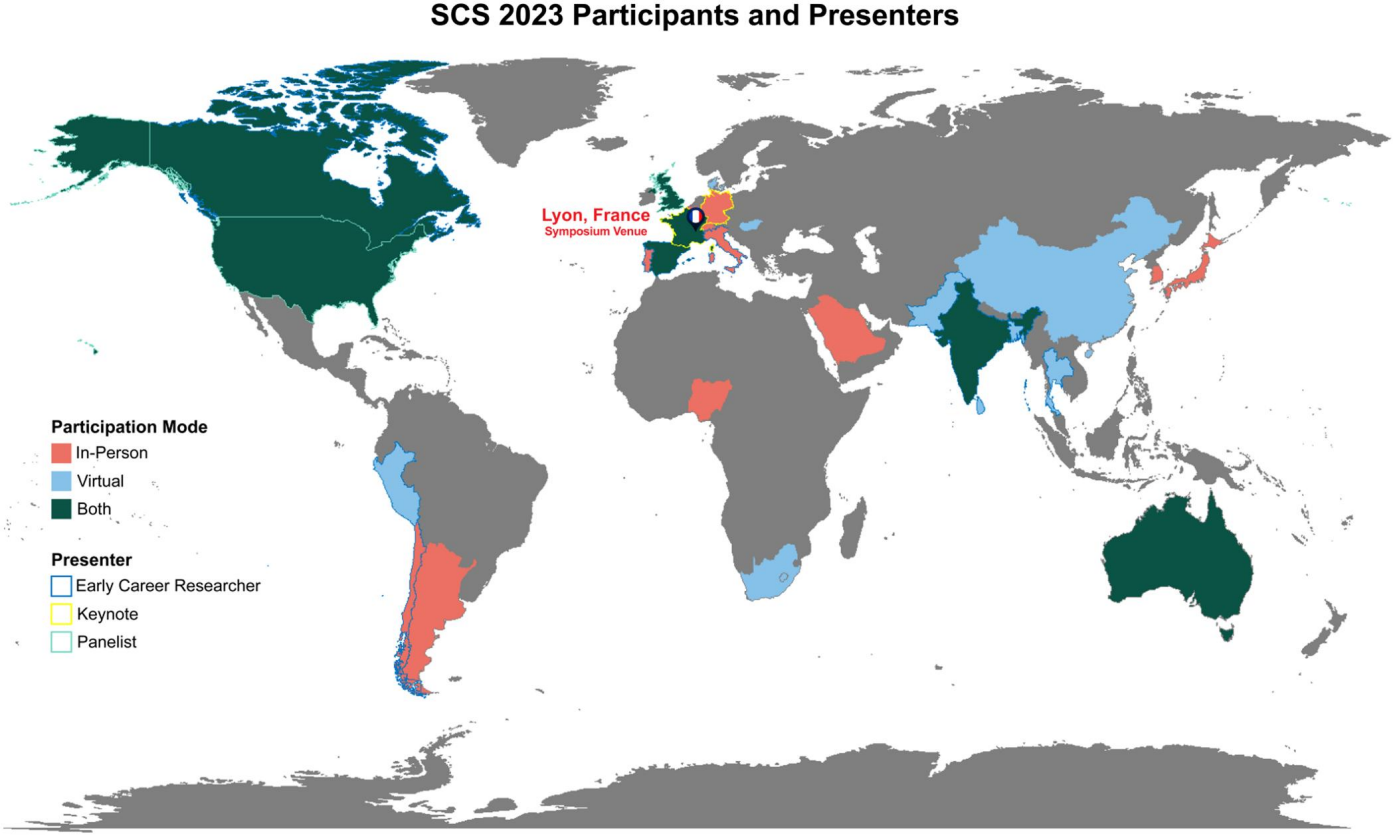
Geographical distribution of symposium participants.

Geographical distribution also played a significant role in shaping the symposium’s landscape. With Lyon, France serving as the symposium’s venue, the conference naturally attracted presenters from European institutions. Notably, most of the participants from European countries were able to travel to the venue for their presentations. In contrast, a majority of presenters from Asia chose the virtual format, with only one Asian participant joining the event in person. Similarly, a majority of the participants from North America also opted for the virtual presentation format. This diversity in participation transcended geographical barriers, providing people from different corners of the globe the opportunity to attend the symposium. Figure 2 represents the geographical distribution of the symposium attendees.

## 3 Keynote talks

**Figure 3.**
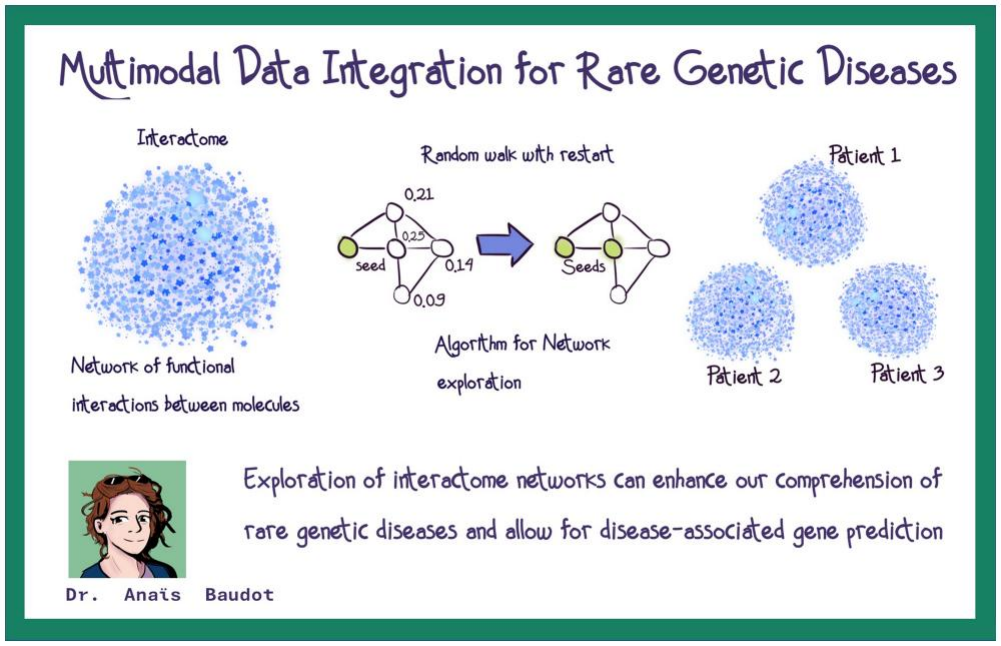
Graphical summary of the keynote speech “Multimodal Data Integration for Rare Genetic Diseases” by Prof. Anaïs Baudot.

The SCS2023 was honored to host two distinguished keynote speakers who imparted invaluable insights to budding scientific minds. Prof. Anaïs Baudot, a Centre National de la Recherche Scientifique (CNRS) director of research in computational and systems biology, sets the tone for the symposium by delving into the realm of systems biomedicine. During the opening keynote, she addressed the audience on “Multimodal Data Integration for Rare Genetic Diseases,” emphasizing the critical role of integrating diverse data types to enhance our understanding of the molecular mechanisms of such diseases. Prof. Baudot also introduced the Random Walk with Restart, an algorithm for network analysis that can also be applied to multiplex and heterogeneous networks for disease-associated gene prediction. A graphical summary of the keynote speech by Prof. Anaïs Baudot is shown in Fig. 3.

**Figure 4.**
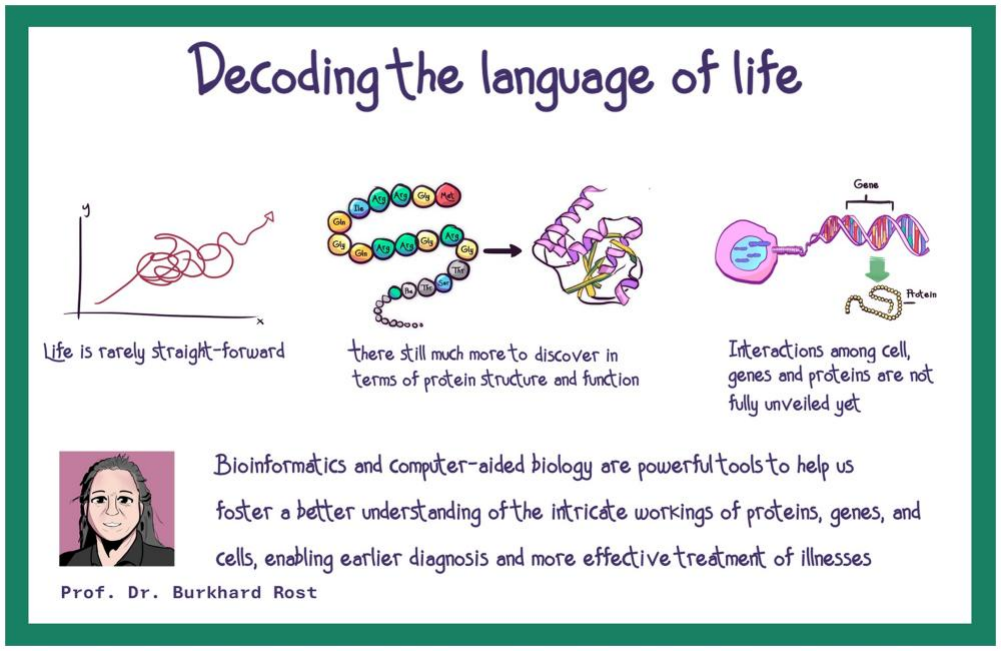
Graphical summary of the keynote speech “Decoding the language of life” by Professor Burkhard Rost.

The afternoon session started with a worldwide known figure of the bioinformatics field, Prof. Burkhard Rost, Chair of Bioinformatics at the Technical University of Munich, providing a captivating exploration of the convergence of artificial intelligence (AI), machine learning, and evolution. As a former ISCB President and a member of the New York Academy of Sciences, Prof. Rost brought a wealth of experience to SCS2023. Prof. Rost captivated the audience with his insights on “Decoding the Language of Life,” aiming to foster a better understanding of the intricate workings of proteins, genes, and cells through advanced computational methodologies. Professor Rost’s keynote speech has been summarized in Fig. 4.

Together, the keynotes underscored the significance of computational and systems biology in addressing real-world challenges and highlighted the symbiotic relationship between biology and advanced computational approaches. Prof. Baudot and Prof. Rost also shared their views on professional development and dispensed precious advice on how to grow as scientists, which will surely be treasured by the young researchers attending SCS2023.

## 4 Presentations of ECRs

### 4.1 Oral presentations

**Figure 5.**
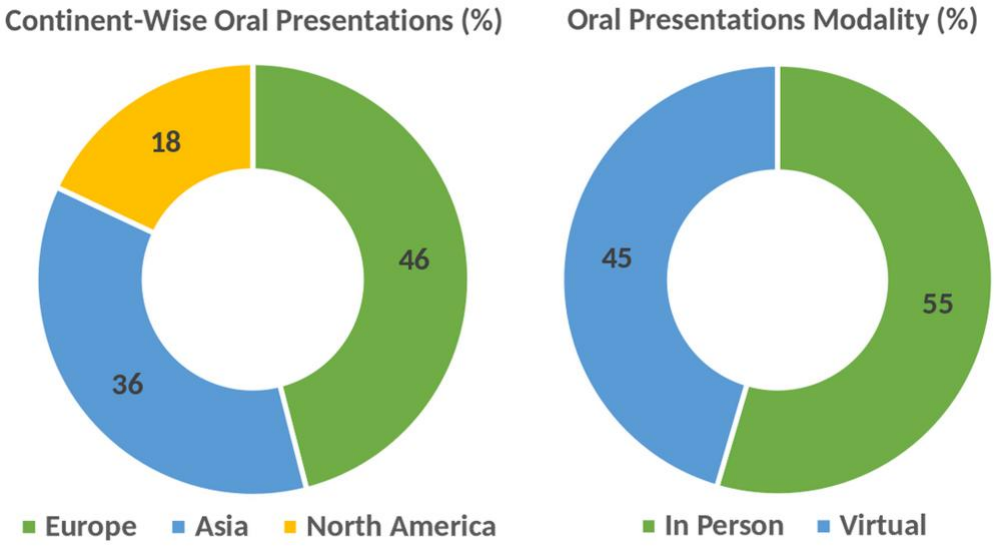
Statistics of affiliated countries and attendance modality of oral talk presenters at SCS2023.

ECRs’ presentations are the core of any ISCB-SC symposium, as they present unique opportunities for researchers to showcase their work in depth while providing the audience with a chance to delve into the intricacies behind their work. SCS2023 featured 11 oral presentations from researchers working in different fields within the fields of computational biology and bioinformatics. Considering the hybrid nature of this year’s symposium, we accepted both in-person and online oral presentations from researchers. There were 6 (55%) presenters who delivered their talks at the symposium venue, while the remaining 5 (45%) joined virtually through our online platform. This balance between in-person and online presentations bears witness to our commitment to making this symposium a successful hybrid event. The statistics for oral presentations of the symposium are presented in Fig. 5.

Each ECR was allotted 10 min to present their work, followed by a 5-min interactive Q&A session. The oral presentations were selected from the abstracts submitted based on a rigorous assessment by a reviewer panel. Authors were also allowed to express their preference for oral or poster presentations while submitting their abstracts. The abstracts receiving the highest scores were selected for oral presentations.

### 4.2 Flash talks

**Figure 6.**
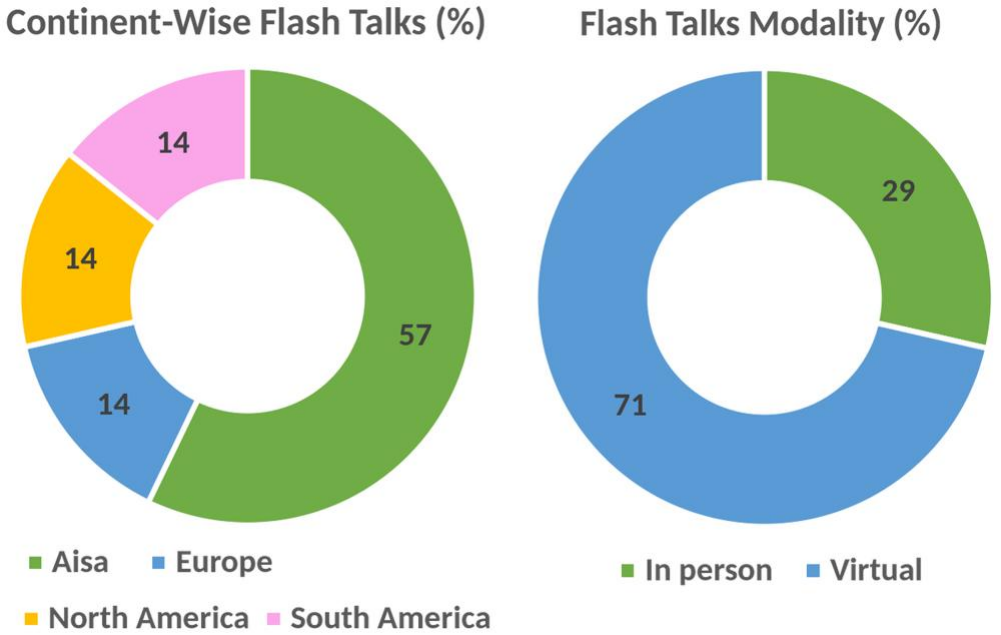
Statistics of the flash talk presenters at SCS2023.

Flash talks are short talks that stimulate extended discussions around poster sessions. These brief yet informative presentations introduce research stories as a lightning bolt of insight, thereby living up to their name. Our SCS program featured seven flash talks of 5 min each with two speakers presenting live from the venue and the remaining five joining via live streaming. Flash talks accounted for a substantial portion, nearly 39%, of all student talks and presentations in our program. These talks not only encouraged the exchange of ideas between speakers and the audience but also played a crucial role in cultivating research collaborations. With a selection rate of 12.5%, flash talks were a competitive and sought-after component of the event. The selection procedure for flash talks involved a rigorous peer review, where the chosen talks demonstrated a compelling edge over traditional poster presentations but a slight mark down from the selected oral presentations. Figure 6 shows the statistics for the flash talks presented in SCS2023.

### 4.3 Categorizing the talks

The speakers selected for oral presentations and flash talks delivered their work within 15 and 5 min, respectively, in different sessions of the SCS2023 program. The poster session was also held both in-person and virtually. The organizing team of SCS2023 categorized the selected and peer-reviewed talks of ECRs into three broad research domain-based categories. The essence of each of these ECR talk sessions and different modes of presentations by the ECRs has been summarized in the following sections.

#### 4.3.1 Session 1: proteins, modelling, and drug design

Following Prof. Anaïs Baudot’s keynote speech, the first session of the symposium centered around peptides and proteins, delving into the analysis of their functions, interactions, and their roles as drug targets. Researchers worldwide have shown significant interest not only in understanding the functions but also in exploring structure–activity relationships and protein–protein interactions. As the functional mediators of biological processes, proteins have always stood out as compelling targets for designing novel therapeutics. During this session, five oral talks and one flash talk were presented by ECRs. The topics covered the analysis of interactions between membrane protein–protein complexes, the development of a bioinformatics pipeline and R package for the identification of drug targets for different types of cancer, and the impact of drugs on the proteomic profiles of extracellular vesicles, among others. These presentations covered a myriad of topics, encompassing prevailing bioinformatics research on proteins and potential applications in medicine.

#### 4.3.2 Session 2: toward the multi-omic era

In recent years, the overwhelming surge in sequencing data has immersed us in a sea of information. To navigate this abundance, targeted efforts to extract meaningful insights involve analysis across various levels. Genomic, transcriptomic, proteomic, and metabolomic approaches converge into multi-omic strategies, and the talks presented in this session collectively propel us “Towards a multi-omic era.” A total of eight talks were presented in this session, including six flash talks and two oral talks. The oral talks depicted complex molecular landscapes underlying prevalent diseases, such as Parkinson’s and cancer, providing insights into potential therapeutics. On the other hand, the flash talks presented a blend of computational advancements and biological relevance. Half of the flash talks explored the applications of multi-omic methodologies across distinct realms of biodiversity, spanning microbial communities to domesticated cotton species along with the viruses at the borders. The remaining flash talks introduced novel methods and tools for functional comparisons of transcriptomics and biomarker discovery. The diverse set of topics covered emphasized the need for integrative approaches in multi-omics to foster breakthroughs in personalized medicine and targeted therapies and to understand the complexities of diseases in an interconnected manner.

#### 4.3.3 Session 3: from cell to genome levels

The final session of the day spotlighted one of the most exciting and novel technologies in biological science, single-cell sequencing. Recent developments have enabled us to study each cell’s genome and transcriptome individually, but they have also brought new challenges. Analyzing the vast and complex data generated through large-scale single-cell sequencing is still a daunting task, and the production of new pipelines and packages is still in active development. Beyond sequence data, research interest extends to the epigenome, the spatial arrangement of the chromatin, and temporal change in expression profiles. This session featured four oral talks, focusing on the development of a bioinformatic pipeline to analyze single-cell RNA-seq data, the exploration of the promoter-enhancer interactome landscape controlled by p53, species-agnostic transfer learning by adapting heterogeneous domain, and more. The session marked the end of the symposium’s presentation segment, offering an extensive discussion on the recent developments in the field of genomic studies.

### 4.4 Poster presentations

**Figure 7.**
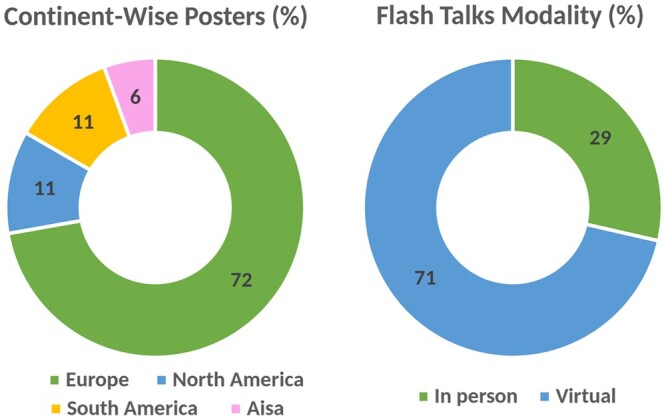
Attendance mode and countries of the poster presenters at the SCS2023.

Poster presentations provide a platform for one-to-one research discussions. At SCS2023, we featured a total of 18 posters. Through the virtual poster hall, SCS2023 opened a soft lounge of interactions and extended discussions over the web. While it may not fully replicate the experience of in-person engagement during live poster sessions, it offered a valuable platform for researchers to share their work with a global audience from the comfort of their homes. A large majority of poster presentations at SCS2023 were virtual with a notably high number of participants from Europe. The statistics are presented in Fig. 7.

All oral presentations, flash talks, and poster presentations covered a diverse range of research topics, including neurodegenerative diseases, cancer, imaging and biomarkers, CRISPR screen datasets, and multi-omics methods.

### 4.5 Best presentations awards

The Best Oral Presentation award was presented to Giulia Cesaro from the University of Padova, Italy, for presenting “CClens: a cellular communication workflow for large-scale single-cell RNA sequencing data.” This talk was voted the best in this category by the participants of the symposium. Nikhil George from the University of Waterloo, Canada, received the Best Flash Talk Award for his presentation entitled “Virus-host interactions in a municipal landfill include non-specific viruses, hyper-targeting, and interviral conflicts.” The winning poster at SCS2023, titled “Prediction of bacterial interactomes based on genome-wide coevolutionary networks: an updated implementation of the Context Mirror approach,” was presented by Miguel Fernandez Martin from the Barcelona Supercomputing Centre.

Considering that all winners were in-person presenters and based on our experiences in past hybrid events, we noticed that in-place discussions may provide more impactful interactions with the audience than in digital settings. This facilitated the exchange of ideas between the presenters and the audience, allowing more effective science communication.

## 5 Round table discussion: “Exploring the potential of AI in revolutionizing bioinformatics research: Opportunities and challenges”

With the rise of cutting-edge AI tools, such as ChatGPT (Ray 2023), the interest in AI has spread among the general population, extending beyond the scope of computational scientists. Amid this AI frenzy, groundbreaking tools have emerged within the field of computational biology. A notable example is AlphaFold 2, which accurately predicts the 3D structure of proteins from their amino acid sequences. However, AI’s influence transcends structural biology as novel tools continue to emerge and enrich the field of computational analysis.

In light of this AI revolution, we made a deliberate choice to center the discussion at the SCS2023 Roundtable on exploring the potential of AI in revolutionizing bioinformatics research. Our focus was particularly on uncovering the opportunities and challenges that these AI tools can bring to the scientific community. To facilitate insightful discussions, we assembled a panel of experts in the field. Our distinguished panelists included Alex Bateman, a computational biologist and Head of Protein Sequence Resources at the European Bioinformatics Institute, UK; Lucia Peixoto, an associate professor in the Department of Translational Medicine and Physiology at Washington State University, USA; Dan DeBlasio, an assistant professor of Computer Science at the University of Texas at El Paso, USA; and Farzana Rahman, an assistant professor in Computer Science at Kingston University, London, UK.

**Figure 8.**
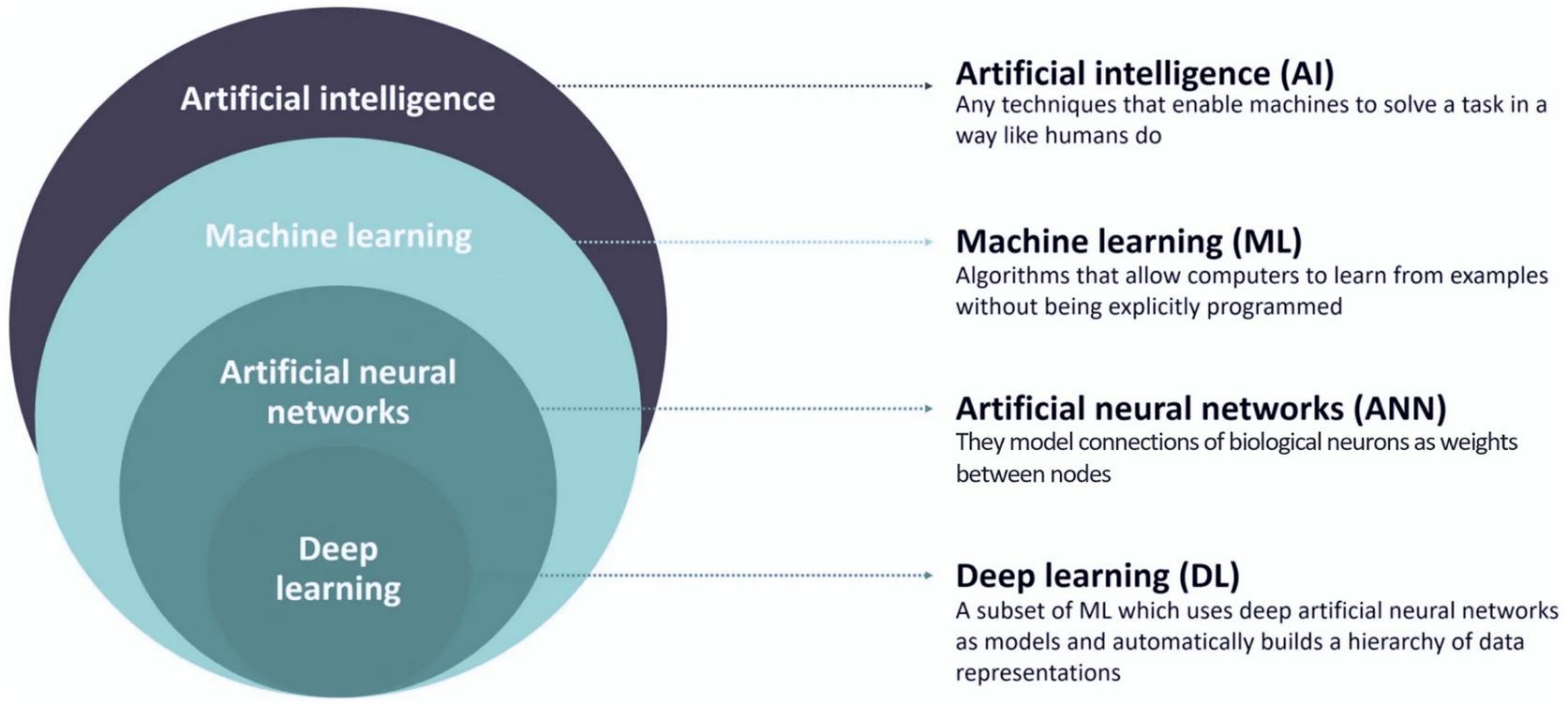
Definitions of popular AI terms. Adapted from HPE and BBN times.

The structure of our session was modeled after the United Nations’ format for roundtable discussions, adapted for a 1-h duration. To select the pertinent topics for discussion, we distributed a Google form among attendees and the general public through social media channels. Following this inquiry, we identified the following topics: (i) Impact of AI in Bioinformatics and the development of novel applications; (ii) ethics involving data management; (iii) the biological questions AI may solve in the future; and (iv) concerns about AI replacing the labor of human bioinformaticians. To ensure that all attendees were on the same page regarding the various AI terminologies, the session began with a brief introduction by moderator Estefania Torrejón. This introduction clarified distinctions among terms, such as AI, machine learning, deep learning, and neural networks. Figure 8 shows a brief description of these popular AI terms.

The first question posed to the panelists prompted them to introduce themselves and share how the AI revolution had impacted their respective research areas. While their responses varied, all panelists embraced AI systems as valuable tools for computational biology. Regarding the ethics of data management, it was emphasized that AI handling data, in and of itself, possesses no ethical implications. Ethical questions arise from how data are collected, transferred, and utilized, but these concerns extend beyond AI and encompass data management in general. Moving on to the third question, our panelists were encouraged to exercise their imagination and envision the biological questions that AI might resolve in the near future. One particularly intriguing response highlighted AI’s potential to unravel the intricate mechanisms underlying brain function, knowledge that could help us tackle various neurological disorders. The final question addressed a prominent topic within the community: whether AI has the potential to replace human bioinformaticians. The consensus was that, even if our roles change in the future, scientists will still be essential for formulating questions, designing studies, and determining whether results support hypotheses. AI systems may assume certain tasks, but human oversight will remain crucial.

Finally, attendees had the opportunity to pose specific questions to our panelists, including inquiries about AI’s usage as a literature source or co-author of scientific papers. The consensus was that there is no harm in employing these innovative tools for good, as long as ethical standards and trust within the scientific community are upheld.

The key takeaway from this session is that AI systems are tools that can enhance our work as computational biologists. However, maintaining transparency in scientific endeavors, even when assisted by AI, remains our ethical duty as scientists.

## 6 Social event

We are grateful to Harvard Medical School for the generous sponsorship, which allowed us to organize a social event close to the main conference venue for our attendees.

This informal gathering provided a conducive atmosphere and inclusive environment where young bioinformaticians and computational biologists had the opportunity to engage in discussions regarding science, aspirations, dreams, and passions. Moreover, it served as a platform for the exchange of ideas, facilitating the convergence of researchers from various disciplines, and fostering substantive dialogues among individuals with similar research backgrounds. As organizers, we were elated to witness the event venue brimming with peers, and—even more gratifying—to observe esteemed senior researchers joining us after the main conference to interact with the students. These interactions revolved around ongoing research, future projects, ambitions, and the invaluable sharing of past experiences.

Among our distinguished guests, we had the privilege of meeting Nils Gehlenborg, Associate Professor of Biomedical Informatics at Harvard Medical School, who represented our esteemed sponsor. He shared insights into the history of the Student Council Symposia, recounting their beginning and their enduring tradition. For instance, he described the genesis of the SCS logo, which was conceived nearly two decades ago and continues to serve as an emblem of our enduring commitment to advancing scientific discourse and nurturing the next generation of bioinformaticians and computational biologists.

## 7 Discussions

### 7.1 Challenges and adaptations

Hosting the first hybrid and 19th ISCB Student Council Symposium came with its fair share of challenges, particularly as it marked the transition back to an in-person format after several years of virtual events due to the COVID-19 pandemic. Navigating this shift required meticulous planning to ensure the safety and satisfaction of all participants. Here are some key challenges encountered and the corresponding adaptations.

#### 7.1.1 Shifting to in-person format

The decision to return to an in-person format was met with excitement, but it also entailed logistical challenges. Ensuring compliance with health and safety guidelines, securing a venue for the social gathering, and coordinating travel arrangements were all critical aspects of this transition.

#### 7.1.2 Poster presentation challenges

Poster sessions are an essential component of scientific symposia. SCS2023 successfully managed to adapt these sessions for both in-person and virtual attendees. However, challenges arose in providing a seamless experience for virtual participants, such as finding innovative ways to display posters and facilitate online discussions.

#### 7.1.3 Challenges in virtual presentations

As a hybrid event, accommodating a significant number of virtual participants presented its own set of challenges. Sometimes, in-person participants could not hear the virtual live presentations properly due to sound or internet issues. Ensuring that all attendees, regardless of their mode of participation, had equitable access to content and opportunities for engagement was a top priority.

#### 7.1.4 Audio issues faced by virtual participants

While the virtual format offered global accessibility, it became apparent that some virtual participants encountered audio-related challenges during sessions. These issues, such as background noise, audio disruptions, or unclear audio quality, affected the overall experience for virtual attendees. Addressing audio quality and providing technical support for virtual participants will be a focal point for improvement in future events.

#### 7.1.5 International travel challenges

The symposium’s international scope introduced additional hurdles, including difficulties with international travel. These challenges were exemplified by the inability of one of the chairs to attend in person due to international travel restrictions. This underscores the complexities of international travel permissions for researchers and the need for flexible arrangements in the face of travel barriers. Such restrictions significantly impact our possibilities to provide equal access to our members to in-person events.

#### 7.1.6 Lessons learned

As expected from previous experiences (Mishra *et al.* 2014, Hassan *et al.* 2018, Parisi *et al.* 2019), while SCS2023 was a resounding success, there were undoubtedly valuable lessons learned from the experience. Identifying and addressing any hiccups that arose during the event, including audio issues for virtual participants and gathering feedback from attendees will help pave the way for future iterations of the symposium.

#### 7.1.7 Low African countries participation

Despite having a global reach with attendees from six continents, it is noteworthy that participation from African RSGs was disproportionately low, accounting for only around 1% of total attendees. This disparity highlights the need for accessibility and outreach efforts to engage more researchers from underrepresented regions.

#### 7.1.8 Payment challenges

The registration process for SCS2023 posed challenges for participants (specially the students) from Bangladesh, where many members of the Organizing Team are located, due to the limited availability of international payment options in the country. Similar to the experience reported during the ISCB first Asian Student Council Symposium (Grover *et al.* 2023), participants from this country encountered difficulties making payments through the ISCB platform. To overcome this obstacle, participants sought assistance from friends and family who allowed international transactions or reached out to ISCB directly for separate payment arrangements. This highlights a systemic issue with international payment accessibility in certain regions, and organizers need to be cognizant of such challenges in future event planning.

### 7.2 Observations and trends

One noteworthy observation from SCS2023 was the predominant focus on particular research fields, such as single-cell analysis. The abundance of abstract submissions and presentations in these specific areas reflects the evolving landscape within the field of Computational Biology and Bioinformatics. It signals the community’s keen interest in exploring new frontiers and harnessing the potential of cutting-edge technologies and methodologies.

Additionally, the clues gained from understanding attendee backgrounds and preferences can serve as a compass for future program planning and content selection, ensuring that the evolving needs of computational biologists and bioinformaticians are effectively met.

## 8 Conclusions

The 19th ISCB Student Council Symposium, as the first ISCB-SC global event after the COVID-19 pandemic, can be considered a resounding success, marked by its innovative hybrid format, distinguished keynote speakers, engaging roundtable discussions, and a rich array of diverse presentations. The return to an in-person format after the disruptions caused by the global pandemic was met with enthusiasm and symbolized a rekindling of face-to-face scientific exchange among researchers and students.

SCS2023’s hybrid approach, integrating both in-person and virtual elements, maximized inclusivity and accessibility for a global audience. It allowed attendees to tailor their experiences based on their preferences and schedules, ensuring that scientific discourse remained vibrant and accessible, regardless of geographical constraints.

The symposium’s engaging roundtable discussion on the potential of AI in revolutionizing bioinformatics research highlighted the community’s interest in this transformative field. The valuable insights shared by panelists underscored the importance of AI as a tool to enhance the work of computational biologists while emphasizing the ethical responsibility of scientists concerning its applications.

Moreover, SCS2023 celebrated outstanding research contributions through awards for the best poster, best flash talk, and best oral talk, spotlighting the talent and innovative minds within the community.

The difficulties faced by some participants in making registration payments highlighted the need for increased flexibility in payment options for participants outside of Europe or North America. Organizers must acknowledge and address these challenges to ensure equitable access to scientific events globally. Lessons learned from both ASCS2023 and SCS2023 emphasize the importance of considering the diverse financial landscapes of participants and implementing solutions to facilitate smoother registration processes for attendees from regions with limited international payment options. These experiences serve as a valuable reminder for organizing teams to proactively address such issues, contributing to the overall inclusivity and success of future symposiums and events.

As SCS2023 successfully bridges the virtual and physical realms of scientific collaboration, it sets a benchmark for future symposia and conferences in Computational Biology and Bioinformatics. The symposium’s adaptability, commitment to inclusivity, and aim to foster connections among the upcoming generation of computational biologists show the resilient and dynamic qualities of this scientific community. With each passing year, SCS continues to nurture and empower the future leaders of the field, ensuring that the spirit of innovation and discovery remains at the forefront of Computational Biology and Bioinformatics.

## Data Availability

The data underlying this article are available in Zenodo at https://doi.org/10.5281/zenodo.8173977.
